# Digital Therapeutic Care and Decision Support Interventions for People With Low Back Pain: Systematic Review

**DOI:** 10.2196/26612

**Published:** 2021-11-19

**Authors:** Daniel Lewkowicz, Tamara Slosarek, Sarah Wernicke, Antonia Winne, Attila M Wohlbrandt, Erwin Bottinger

**Affiliations:** 1 Digital Health Center Hasso Plattner Institute University of Potsdam Potsdam Germany; 2 Hasso Plattner Institute for Digital Health at Mount Sinai Icahn School of Medicine at Mount Sinai New York City, NY United States

**Keywords:** digital therapeutic care, decision support interventions, low back pain, behavior change techniques, back, orthopedic, systematic review, digital therapy, decision support, mobile phone

## Abstract

**Background:**

Low back pain (LBP) is the leading cause of worldwide years lost because of disability, with a tremendous economic burden for health care systems. Digital therapeutic care (DTC) programs provide a scalable, universally accessible, and low-cost approach to the multidisciplinary treatment of LBP. Moreover, novel decision support interventions such as personalized feedback messages, push notifications, and data-driven activity recommendations amplify DTC by guiding the user through the program while aiming to increase overall engagement and sustainable behavior change.

**Objective:**

This systematic review aims to synthesize recent scientific literature on the impact of DTC apps for people with LBP and outline the implementation of add-on decision support interventions, including their effect on user retention and attrition rates.

**Methods:**

We searched bibliographic databases, including MEDLINE, Cochrane Library, Web of Science, and the Physiotherapy Evidence Database, from March 1, 2016, to October 15, 2020, in accordance with the PRISMA (Preferred Reporting Items for Systematic Reviews and Meta-Analyses) guidelines and conducted this review based on related previously published systematic reviews. Besides randomized controlled trials (RCTs), we also included study designs with the evidence level of at least a retrospective comparative study. This enables the consideration of real-world user-generated data and provides information regarding the adoption and effectiveness of DTC apps in a real-life setting. For the appraisal of the risk of bias, we used the Risk of Bias 2 Tool and the Risk of Bias in Non-Randomized Studies of Interventions Tool for the RCTs and nonrandomized trials, respectively. The included studies were narratively synthesized regarding primary and secondary outcome measures, DTC components, applied decision support interventions, user retention, and attrition rates.

**Results:**

We retrieved 1388 citations, of which 12 studies are included in this review. Of the 12 studies, 6 (50%) were RCTs and 6 (50%) were nonrandomized trials. In all included studies, lower pain levels and increased functionality compared with baseline values were observed in the DTC intervention group. A between-group comparison revealed significant improvements in pain and functionality levels in 67% (4/6) of the RCTs. The study population was mostly homogeneous, with predominantly female, young to middle-aged participants of normal to moderate weight. The methodological quality assessment revealed moderate to high risks of biases, especially in the nonrandomized trials.

**Conclusions:**

This systematic review demonstrates the benefits of DTC for people with LBP. There is also evidence that decision support interventions benefit overall engagement with the app and increase participants’ ability to self-manage their recovery process. Finally, including retrospective evaluation studies of real-world user-generated data in future systematic reviews of digital health intervention trials can reveal new insights into the benefits, challenges, and real-life adoption of DTC programs.

## Introduction

### Background

Low back pain (LBP) is the leading cause of worldwide years lost because of disability, with a global point prevalence of 9.4% and a reported lifetime prevalence of up to 84% [1,2]. Moreover, LBP is responsible for most absences from work as well as productivity losses, which ultimately results in a tremendous societal and economic burden [3]. Current clinical guidelines recommend a multimodal treatment approach for people with nonspecific, nonacute LBP, including remaining physically active, exercising and receiving educational therapy, and using psychosocial interventions [4,5].

Digital therapeutic care (DTC) programs provide a scalable, universally accessible, and low-cost approach to deliver these key components of a multimodal treatment. Using smartphone or browser-based apps, people with LBP can proactively self-manage their recovery process through remote physical and mindfulness exercises and in-depth explanatory educational material. Initial research investigating a DTC app to self-manage LBP has shown an overall positive effect on pain levels and functional disability [6]. In this virtually unsupervised approach, motivational factors, coping behavior, and self-management abilities play a critical role in patient literacy and empowerment with regard to adherence to the treatment program [7]. Thus, novel add-on personalized decision support interventions provide the possibility of guiding the user through the program and achieving sustainable behavior change through, for instance, tailored feedback messages, push notifications, and data-driven activity recommendations [8]. However, the benefits of a DTC program with add-on decision support interventions remain unclear and require further investigation [9].

Moreover, low user retention and high attrition rates are unresolved challenges, with reported nonengagement levels of up to 70% [10,11]. In this regard, user retention describes the adherence to, and overall response rate of, the DTC program [12]. This involves the sustained use of individual treatment modules. Engagement in the program can be measured, for instance, by the number of completed exercises or the time spent on the educational material [11]. Alternatively, the attrition rate focuses on the dropout of participants and, thus, their discontinuation of the DTC program [13]. In the treatment of people with LBP, both user retention and attrition rate play a critical role in understanding the causal dependencies with regard to the long-term impact of digital therapeutic interventions.

Previous systematic reviews focused on investigating the impact of DTC apps or decision support interventions in a controlled clinical trial–based environment, which determines the efficacy of the intervention under considerably ideal conditions [14]. In contrast, the intervention’s effectiveness provides information on health-related outcomes in a real-world setting from people using the app either on their own initiative or after receiving a physician’s prescription. Evidence regarding the difference in outcomes between a controlled trial setting and real-world use is lacking because previous systematic reviews only included randomized controlled trials (RCTs) because they represent the gold standard [9,14,15]. However, in future data-driven research on digital health interventions, retrospective evaluations could generate new insights into the effectiveness and engagement of DTC programs. In fact, the quickly evolving regulatory environment in favor of digital ecosystems advocates research platforms and databases to facilitate the evaluation of real-life user data. Finally, Germany’s newly introduced Digital Healthcare Act allows the reimbursement of the cost of digital health apps by the statutory health insurance providers once the app is listed in the Digital Health Applications directory [16,17]. For this purpose, manufacturers are obliged to provide scientific evidence in the form of at least retrospective comparative studies proving that their digital health app yields positive health care effects [16]. This approach directly enables the consideration of real-life user-generated data and provides information regarding the adoption and effectiveness of the digital health app in a real-world setting.

### Related Work

Various systematic reviews have elaborated on the impact of digital therapeutic interventions for people with LBP [9,15]. Nicholl et al [9] performed a comprehensive review with the most substantial overlap to our research question investigating digital support interventions for the self-management of LBP. Their work is part of the European Union (EU)–funded selfBACK project, which aims to develop an app that provides tailored, algorithm-based digital decision support interventions for the self-management of LBP [18]. The authors identified 6 completed RCTs but could not conclude under what circumstances which type of digital support intervention was effective for people with LBP. Because of the variability of study interventions and the homogeneous participant cohorts, which consisted predominantly of White, well-educated, and middle-aged women, it became clear that further studies are necessary to evaluate the benefits of digital support interventions for broader populations.

In a more recent review, Hewitt et al [15] investigated the impact of digital health interventions in a broader context of musculoskeletal conditions. In their review, the authors included 19 studies, of which 9 reported statistically significant reductions in musculoskeletal pain and 10 reported statistically significant improvements in functional disability. However, because of the consideration of predominantly stand-alone interventions and missing relatedness to LBP specifically, a recent systematic literature review dedicated to a holistic DTC program is, to the best of our knowledge, currently lacking.

It is worth mentioning that 2 systematic reviews have investigated apps that aim to support people with LBP with self-management, monitoring, or decision support interventions and are available on the iTunes and Google Play stores [19,20]. The first review, from 2017, found 61 smartphone apps, whereas the more recent one, from 2020, identified 74 apps available to download for smartphone users. The high and still increasing number of smartphone apps also underlines the need for an updated review from a scientific, clinical trial–based perspective.

### Objective

The aim of this review is to evaluate recently published clinical evidence regarding the efficacy and effectiveness of digital therapeutic interventions for people with LBP. Moreover, we seek to synthesize the characteristics and components of the respective digital therapeutic programs, the type of delivery and interactivity with the user, and the extent of the deployed decision support interventions. Thereby, we aim to extract overall retention and attrition rates of the therapeutic care apps and summarize how current decision support interventions contribute to overall engagement levels and possibly influence health-related outcome measures.

## Methods

### Study Design

Following the PRISMA (Preferred Reporting Items for Systematic Reviews and Meta-Analyses) statement, we performed a systematic literature review to identify and analyze recent scientific evidence regarding digital therapeutic and decision support interventions for people with LBP [21]. Notably, this systematic review was not preregistered in an international prospective registry such as PROSPERO. The new field of DTC apps and decision support interventions for LBP has rapidly emerged in scientific research over the past years. New nomenclature has arisen from ongoing software implementations and the increase in the number of innovational digital therapy features. These developments required an explorative approach to defining the inclusion and exclusion criteria for a profound systematic review to ensure that all relevant studies could be included. Therefore, we chose a snowballing search method and, subsequently, extended our ongoing search to a systematic review. Nonetheless, being aware of potential biases that may result from the lack of a prospective preregistration, we have presented our findings using a narrative approach, with the primary goal of summarizing recent technological improvements and implications in the field of digital therapy for LBP.

### Search Strategy

We searched the bibliographic databases (1) MEDLINE through PubMed, (2) Cochrane Central Register of Controlled Trials in the Cochrane Library, (3) Web of Science Core Collection, and (4) the Physiotherapy Evidence Database and included English- and German-language literature published in peer-reviewed journals. In addition, we screened the reference lists and tracked the citations of all included studies for eligibility.

This review’s search concept is based on 2 main pillars: (1) LBP and (2) digital therapeutic and decision support interventions. These search terms were extended with specific terminology and synonyms using Boolean operators and the respective Medical Subject Headings and are aligned with the updated method guideline for systematic reviews provided by the Cochrane Back and Neck group [22]. The detailed search queries for the corresponding databases are presented in Multimedia Appendix 1.

The final search was conducted on October 15, 2020. All collected studies were saved in a reference management software program, and duplicates were removed. In the first iteration, the titles and abstracts of the remaining studies were screened by 2 reviewers (DL and AMW) independently. Any disagreements would lead to the inclusion of a study for full-text screening. Subsequently, full-text screening was also conducted by 2 independent reviewers (DL and AMW). This time, the studies on which the reviewers disagreed were assessed for eligibility by a third reviewer (SW) and resolved through discussion.

### Inclusion Criteria

We have summarized our inclusion and exclusion criteria in Textbox 1. In brief, we included all publications, with the primary aim of investigating the efficacy or effectiveness of a multidisciplinary DTC program with respect to health-related outcomes for people with LBP. Furthermore, our presearch and small pilot review of related work showed that prior systematic reviews had evaluated our research questions or comparable ones before 2016. Therefore, our systematic review complements the benchmark work of Nicholl et al [10], who have adequately elaborated the time frame until March 2016; therefore, we have included published studies from March 1, 2016, to October 15, 2020. Our approach is underpinned by our focus on the significant technological improvements in the field of decision support interventions as a new feature in DTC apps that have become available in recent years. Because of these emergent advancements and the changing terminology, the continuation of, and comparison with, the work of Nicholl et al [10] are not within the scope of this review.

**Inclusion and exclusion criteria according to the population or patient problem, intervention, control, and outcomes (PICO) concept.**

**Inclusion criteria**

- Population: People aged >16 years with low back pain.
- Intervention: Any interactive digital and internet-based (health) app that provides digital treatment therapy through an electronic device, that is, computer, tablet, or smartphone. Digital treatment includes access to a digital exercise program, including exercise instructions (eg, video-guided). Moreover, the app contains at least one intervention that addresses the biopsychosocial factors of low back pain, for example, through digital educational material or a digital psychological intervention in the form of cognitive behavioral therapy, or enables self-management, for example, through digital decision support interventions.
- Control: Treatment as usual or any other nondigital form of therapy regarding exercises and educational material for people with low back pain or older versions of the investigated digital therapeutic app or baseline measures.
- Outcomes: Any health-related primary outcome measure that is related to pain or functional disability. Secondary outcomes might include psychological factors (eg, depression), physical activity, medication use, health care resource use, health care costs, or digital therapy program adherence and retention rates.
- Study design: Randomized and nonrandomized controlled trials (including pilot randomized controlled trials); observational analytical studies, either prospective or retrospective; or intraindividual single-arm comparison studies.

**Exclusion criteria**

- Patient problem: Unspecified chronic pain or other musculoskeletal disorder conditions, for example, neck or knee pain.
- Intervention: Digital health apps using a fully automated text-based health care chatbot; smartphone-based standing posture, sitting posture, or range-of-motion recording or human activity recognition; self-referral decision support interventions; smartphone use only for a 6-minute walking test; internet interventions that include only a reminder or pain monitoring or reporting systems; stand-alone digital cognitive functional therapy; exercise therapy through DVD, CD, or a console, for example, Nintendo Wii; or other website-based interventions.
- Study design: Observational, purely descriptive studies, for example, cross-sectional, qualitative, mixed methods, acceptability, or development studies.

In our review, we also included study designs with an overall lower scientific evidence level than RCTs of at least retrospective comparative studies for the following reasons: first, because of Germany’s newly introduced Digital Healthcare Act, German manufacturers of digital health apps are obliged to provide scientific evidence in the form of at least retrospective comparative studies proving that their app yields positive health care effects [16,17]. Therefore, we adopted this selection criterion of scientific evidence for this review to elaborate on the feasibility of a framework that considers real-world evidence for regulatory decisions.

Second, although RCTs remain the gold standard for providing the highest clinical evidence, the optimal control conditions in digital health intervention trials require further investigation [23]. Choosing treatment as usual as the control group in prospective RCTs might lead to a so-called “app-physician competition bias” [23]. The physicians’ awareness of the controlled study design, for example, when competing against a digital therapeutic app for LBP, may cause them to update their knowledge regarding the newest guidelines and treatment recommendations. Thus, the consideration of divergent control groups and retrospective, cohort study designs might be useful for digital therapeutic apps, which will be evaluated with regard especially to the number of associated biases and confounders.

### Data Synthesis

Data of all included studies were extracted by 2 independent reviewers who were randomly selected from a pool of 5 reviewers (DL, AW, TS, SW, and AMW) for each included study regarding the following outcomes: characteristics of included studies, characteristics of the participants, characteristics and components of the digital therapeutic interventions as well as retention rates, and data related to primary and secondary outcome measures. Because of the heterogeneity of the included studies, it was not feasible to conduct a meta-analysis. Despite making assumptions of the apparent similarity of most of the included studies in this review, we decided not to conduct a statistical meta-analysis because it could further compound possible biases regarding meaningful clinical recommendations and is therefore not justified. We have included a broad range of different DTC apps to narratively describe the progress made in this enormously increasing field of digital health. Our primary goal of following a narrative approach in the data synthesis is to provide information to researchers, manufacturers, and decision-makers on the status of scientific research in DTC. Thus, we focused on creating an overview of recent technological improvements, for example, decision support interventions that accompany digital therapy for people with LBP. Because of this focus, we did not extensively narrow the inclusion and exclusion criteria concerning the study design, that is, the time frame of follow-up measures, the comparator group, or the outcome measurements, including different tools and scales. Moreover, combining only a subgroup of our review’s included studies into a meta-analysis would potentially have led to misleading conclusions, especially because we have only included studies published from March 1, 2016, to October 15, 2020.

### Quality Appraisal

For the assessment of the methodological quality of the included studies, we used 2 separate tools to adequately elaborate on the RCTs as well as the observational studies [24]. We chose the Risk of Bias 2 (RoB 2) Tool for assessing “risk of bias in randomized trials” [25], which is based on an earlier version of the Cochrane Collaboration tool for assessing the risk of bias in randomized trials [26], and the Risk of Bias in Non-Randomized Studies of Interventions (ROBINS-I) Tool for assessing the risk of bias in observational studies [27]. Quality assessment was performed independently for each included study by 2 reviewers who were randomly selected from a pool of 5 reviewers (DL, AW, TS, SW, and AMW) for each included study. Studies on which the reviewers disagreed were assessed by a third reviewer (TS or SW) independently and resolved through discussion.

## Results

### Search Results

**Figure 1:**
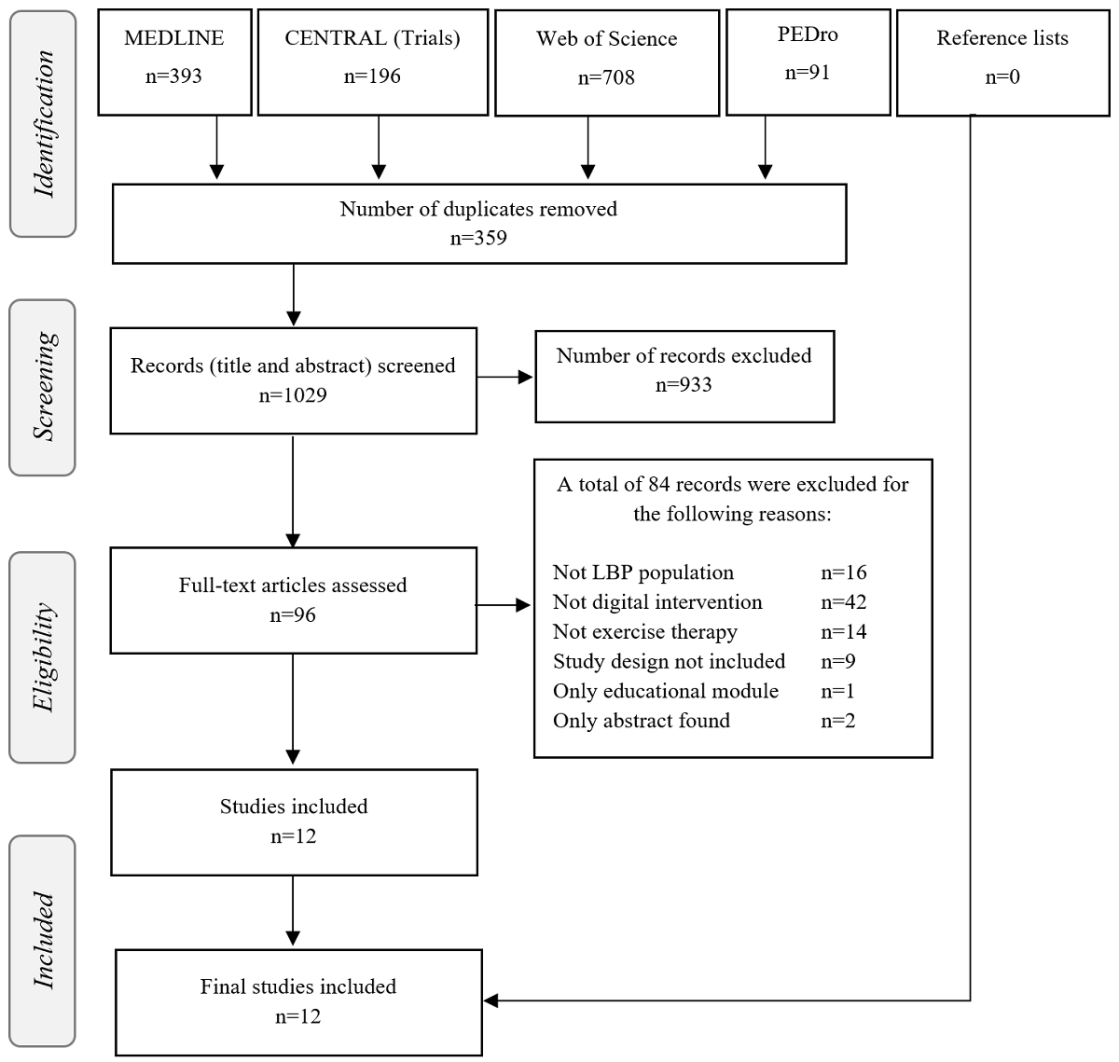
PRISMA (Preferred Reporting Items for Systematic Reviews and Meta-Analyses) flow diagram of the search process (N=1388). CENTRAL: Cochrane Central Register of Controlled Trials; LBP: low back pain; PEDro: Physiotherapy Evidence Database.

We retrieved 1388 citations in total, and after removing 359 duplicates, we screened 1029 publications that were potentially eligible for inclusion in this review. Of the 1029 studies, 96 remained after title and abstract screening for full-text assessment. In the end, of these 96 studies, we included 12 in this systematic review. No additional publications were identified by screening the reference list or Google Scholar’s *Cited by* option of included studies. The iterative steps of our literature search and the reasons for excluding several studies are shown in a PRISMA flow diagram (Figure 1).

### Description of Included Studies

Of the 12 studies, 6 (50%) [28-33] were RCTs and 4 (33%) [34-37] had a retrospective cohort design, whereas the remaining 2 (17%) were a retrospective evaluation [38] and a prospective single-arm trial [39], respectively. Of the 12 studies, 5 (42%) were published in 2020 [28,33,34,37,39], 3 (25%) in 2019 [29-31], 3 (25%) in 2018 [32,36,38], and 1 (8%) in 2017 [35]; moreover, 2 (17%) each were conducted in the United States [30,34], Germany [28,31], and China [29,38], 1 (8%) in India [32], and 1 (8%) in Jordan [33], whereas the remaining 3 (25%) were conducted in multiple countries. Of these 3 studies, 1 was conducted in Denmark and Norway [39] and 1 included participants from Germany, Austria, and Switzerland [35]. In addition, a follow-up study that used the same DTC app was conducted in the United States and the United Kingdom [36]. Of the 12 studies, in 1 (8%), it was not clearly stated from which country the users signed up for the program [37]. Regarding the names of the projects or apps, in 42% (5/12) [28,31,35-37] of the studies, the *Kaia* app was investigated; in 17% (2/12) [30,34], the *Hinge Health* app was investigated; whereas the *selfBACK* app [39], *Snapcare* app [32], *Relieve my back* app [33], *Well Health* app [38], and *eHealth program* [29] were investigated in 8% (1/12) each. The study durations with regard to the digital therapeutic intervention did not vary significantly. In 58% (7/12) [28,30-32,34,35,37] of the studies, the intervention was investigated for 12 weeks or 3 months, 17% (2/12) [33,39] had an intervention duration of 6 weeks, 17% (2/12) had an intervention duration of 24 weeks [36] and 24 months [29], whereas in 8% (1/12) [38], the duration was inconsistent and not clearly reported.

### Study Population

The detailed characteristics of the study participants are listed in Table 1. Overall, the reviewed studies included 10,275 participants. The variation in the total number of study participants was significant, ranging from 41 participants in an RCT [33] to 6468 in a retrospective cohort study [34]. In most of the studies, the number of participants ranged from 93 to 180 [29-33,35,38].

**Table 1:** Characteristics of the participants in the included studies (N=12).

Reference	LBP^a^ duration of included participants	LBP diagnosis^b^	Total number of participants	Age (years), mean (SD)	Female (%)	BMI, mean (SD)
Bailey et al [34]	>12 weeks	Self-reported	6468	42.58 (10.91)	48.53	29.76 (7.11)
Priebe et al [28]	<12 weeks	General practitioner	1245; I^c^: 933; C^d^: 312	I: 42.0 (12.4); C: 37.0 (12.6)	I: 65; C: 64	I: 26.5^e^; C: 26.3
Hou et al [29]	Underwent surgery	General practitioner	168; I: 84; C: 84	I: 51.11 (9.54); C: 49.36 (9.52)	I: 57; C: 50	NR^f^
Shebib et al [30]	>6 weeks in the past 12 months	Self-reported	177; I: 13; C: 64	I: 43 (11); C: 43 (12)	I: 37; C: 48	I: 26 (5); C: 26 (4)
Toelle et al [31]	From 6 weeks to 1 year	General practitioner	101; I: 53; C: 48	I: 41 (10.6); C: 43 (11.0)	I: 72.9; C: 67.4	I: 24.4 (3.31); C: 25.4 (4.6)
Chhabra et al [32]	>12 weeks	General practitioner	93; I: 45; C: 48	I: 41.4 (14.2); C: 41.0 (14.2)	NR	I: 23.15 (4.2); C: 23.54 (3.8)
Almhdawi et al [33]	>3 months	Self-reported	41; I: 21; C: 20	I: 40.48 (7.22); C: 41.70 (6.35)	I: 67; C: 60	I: 27 (3.00); C: 35 (3.00)
Lo et al [38]	<3 months	Self-reported	161	—^g^	24.68	NR
Huber et al [35]	<6 weeks: 13.9%; <12 weeks: 12.8%; >12 weeks: 73.3%	Self-reported	180	33.9 (10.9)	58.3	NR
Clement et al [36]	NR	Self-reported	1251; V1^h^: 196; V2^h^: 1055	V1: 34.8 (11.0); V2: 45.6 (11.6)	V1: 58.2; V2: 49.3	NR
Priebe et al [37]	NR	Self-reported	339; V1: 180; V2: 159	V1: 33.9 (10.86); V2: 46.9 (13.10)	V1: 58.33; V2: 43.79	NR
Sandal et al [39]	Any duration	Physiotherapist or general practitioner	51	45.5 (15.0)	58	27.2 (5.5)

^a^LBP: low back pain.

^b^Defines who referred the participant to the study or who diagnosed low back pain.

^c^I: intervention group.

^d^C: control group.

^e^Calculated based on in-study reported height and weight values.

^f^NR: not reported.

^g^Only categorized values were reported: age 18-25 years: 30 users; age 26-30 years: 31 users; age 31-40 years: 56 users; age 41-50 years: 19 users; age 51-60 years: 20 users; age >60 years: 1 user.

^h^Comparison between 2 subsequent app versions: version 0.x (V1) and version 1.x (V2). V1 includes users who signed in before May 1, 2017, and V2 includes users who signed in after that date.

Of the 12 studies, 1 (8%) [33] included only people who were aged between 30 and 55 years by addressing only office workers, whereas the other 11 (92%) included participants aged ≥18 years up to the age of 65 years, 80 years, or without an upper-bound specification. In 50% (6/12) of the studies [28,30-34], the mean age in the intervention group was between 40 and 43 years. The highest reported mean age was 51.11 years [29], and the lowest was 33.9 years [35]. Regarding the sex of the participants, women were overrepresented in 67% (8/12) of the studies, peaking at 72.9%, 67%, and 65% in 38% (3/8) [28,31,33] of these studies. Of the 12 studies, 1 (8%) [34] reported no significant difference in the female-to-male ratio, 1 (8%) [32] did not report any information, and 1 (8%) [38] reported a female rate of only 24.68%. Of the 12 studies, only 7 (58%) [28,30-34,39] reported on the BMI of the included participants. The average BMI values ranged between 23.15 kg/m^2^ [32] and 29.76 kg/m^2^ [28] in the intervention group, including 17% (2/12) [31,32] of the studies with participants with BMI <25 kg/m^2^ (people of normal weight). The ethnicity and comorbidities of participants were not reported in any of the included studies.

### Risk-of-Bias Assessment

The results of the risk-of-bias assessment of the included studies are presented in Tables 2 and 3. We used the RoB 2 Tool for the included RCTs (6/12, 50%; Table 2) and the ROBINS-I Tool for the nonrandomized studies (6/12, 50%; Table 3). In the RoB 2 analysis, the studies were assessed using predefined signaling questions and were accordingly categorized using standardized wording, that is, *low risk*, *some concerns*, or *high risk* of bias. Similarly, in the nonrandomized trials, the risk of bias was judged to be *low*, *moderate*, *serious*, or *critical*.

**Table 2:** Risk-of-bias assessment of included randomized controlled trials (N=6).

Risk of Bias 2 Tool	Bias arising from the randomization process	Bias due to deviations from intended interventions	Bias due to missing data	Bias in measurement of outcomes	Bias in selection of the reported result
Priebe et al [28]	Some concerns	Some concerns	Low	Some concerns	Low
Hou et al [29]	Low	Some concerns	Low	Some concerns	Low
Shebib et al [30]	Low	Some concerns	Low	Some concerns	Low
Toelle et al [31]	Some concerns	Low	Low	Some concerns	Low
Chhabra et al [32]	Low	Low	Low	Some concerns	Low
Almhdawi et al [33]	Low	Some concerns	Low	Low	Low

**Table 3:** Risk-of-bias assessment of included nonrandomized studies (N=6).

Risk of Bias in Non-Randomized Studies of Interventions Tool	Bias due to confounding	Bias in selection of participants	Bias in classification of intervention	Bias due to deviations from intended interventions	Bias due to missing data	Bias in measurement of outcomes	Bias in selection of the reported result
Bailey et al [34]	Low	Serious	Low	Moderate	Low	Moderate	Moderate
Lo et al [38]	Moderate	Serious	Moderate	Moderate	Unclear	Serious	Moderate
Huber et al [35]	Serious	Serious	Moderate	Moderate	Serious	Moderate	Moderate
Clement et al [36]	Serious	Serious	Moderate	Serious	Moderate	Moderate	Moderate
Priebe et al [37]	Serious	Serious	Moderate	Moderate	Serious	Moderate	Moderate
Sandal et al [39]	Moderate	Moderate	Low	Low	Low	Moderate	Low

The overall risk of bias in a study was determined based on the highest level of risk in at least one domain, that is, the study was judged to be at high risk of bias when at least one domain was considered *high*. The RoB 2 Tool encompasses 5 domains, whereas the ROBINS-I tool encompasses 7. Of the 6 RCTs, 6 (100%) were appraised as having low risk or some concerns regarding potential biases, predominantly regarding bias due to deviations from intended intervention and outcome measurement. Notably, of the 6 RCTs, 1 (17%) achieved double-blinding of participants and assessors by providing a *placebo* version of the same app, which included only advice about general nutrition as a control. In the 6 nonrandomized trials, the overall methodological quality was low and associated with a greater risk of bias: 1 (17%) provided sound to moderate evidence for a nonrandomized trial, whereas 5 (83%) exhibited a serious risk of bias and thus have some important problems across domains. The major biases occur because of confounding in the selection of participants and because of missing outcome data. In detail, these include different durations of the observational period between groups; missing or significantly different demographic compositions between groups; a retrospective recall of preintervention outcome measures, for example, pain level; predefined inclusion criteria that consider only users who have already completed a certain number of exercises in the first 2 weeks after registration; or the inclusion of only users of the pro version of an app that costs €9.99 (US $11.56) per month. Bias due to missing data arose when incomplete data were provided, either because of a high attrition rate or because of a fragmentary analysis of an app’s user database.

### Digital Therapeutic Key Components

We have summarized all investigated DTC apps, including their key components, recommended timing and use frequency, and implemented decision support interventions, in Table 4. The DTC apps involved multiple key components that address the clinical guideline–based recommended multimodal treatment for people with LBP. In all included studies, participants had access to in-app exercise therapy either in the form of videos [28-32,34-39] or picture-based instructions [33]. As another key component, educational material was provided in 92% (11/12) of the apps and involved back pain–specific reading material and papers or rehabilitation plans. The third key component comprised psychosocial interventions that address stress and individual behavioral traits that could influence LBP, that is, in the form of cognitive behavioral therapy, personal health and behavioral coaching, or mindfulness and relaxation techniques in 58% (7/12) of the studies [28,30,31,34-37]. The timing and frequency at which the user was required to engage with the app varied between studies, described in detail in Table 4. All DTC programs were fully digital, that is, they were either smartphone-based or browser-based.

**Table 4:** Digital therapeutic components, decision support interventions, user retention and engagement, and attrition rates in the included studies (N=12).

Study, duration	Digital therapeutic components	Recommended timing and frequency	Decision support interventions	Underlying BCTs^a^	User retention and engagement	Attrition rate,^b^ %
Bailey et al [34], 12 weeks	1. Sensor-guided exercise therapy: instructional videos and real-time graphics; 2. Remote personal health coaching and educational papers	Weekly 3 sessions of sensor-guided exercise therapy, 2 educational papers, 3 aerobic exercise activities, and 4 modules based on cognitive behavioral therapy	Peer-group interaction and support through in-app discussion feed; 20-30 participants who each used a discussion forum	Catastrophizing, active coping methods, fear avoidance, goal setting, and health tracking	Per week: Exercise therapy sessions: mean 2.9 (SD 1.46); education sessions: mean 2.2 (SD 1.55); interactions with coach: mean 7.0 (SD 3.09)	27.71^c^
Priebe et al [28], 12 weeks	RE^d^^,^^e^	RE	RE	RE	Physical exercise: mean 23 days^f^; mindfulness: mean 15 days; education: mean 16 days	27.20
Hou et al [29], 24 months	1. Rehabilitation video instructions; 2. Rehabilitation plans; 3. Communication with physicians through the app	Rehabilitation exercise: twice daily, with each session lasting 20 minutes	Daily rehabilitation exercise reports and alerts (prompting the user to return to the system)	Reminder	High^g^: 62.29%; medium: 26.23%; low: 11.48%	28.57
Shebib et al [30], 12 weeks	1. Sensor-guided exercise therapy; 2. Education, cognitive behavioral therapy, and behavioral coaching; 3. Activity and symptom tracking	Weekly 3 sessions of exercise therapy, 3 aerobic activities, 1-2 educational articles, and cognitive behavioral therapy on a subset of weeks	Peer-group interaction and coach support through in-app discussion feed, checklists, and point goals; weekly	Reminder, peer support, and gamification	Users engaging with the program per week: 75%; total number of workouts: mean 35.7 (SD 28.9); educational articles: mean 7.4 (SD 4.4); cognitive behavioral therapy session: mean 1.4 (SD 1.2)	24.2
Toelle et al [31], 12 weeks	1. Physiotherapy and physical exercise; 2. Back pain–specific education; 3. Mindfulness and relaxation	Daily content consists of components 1-3; recommended use 4 times per week; up to 5 exercises per day	Customizable reminders, push notifications, health coach (chat function)	Reminder and motivation	Kaia app was used on mean 35 days^f^ (SD 22)	20.07
Chhabra et al [32], 12 weeks	1. Tailored home exercise program, including back and aerobic exercises; 2. Activity and health plan	Daily: 4-km walk at a single stretch and 2 sets of 7 back exercises	Daily notifications and reminders; rewards system: points for each milestone achieved and access to the next level once enough points were collected	Gamification and reminder	NR^h^	2.15
Almhdawi et al [33], 6 weeks	1. Set of stretching and evidence-based strengthening exercises; 2. Educational short posts modified from the Back Book	Weekly 3-4 sessions, each session lasting 20 minutes	Daily notifications (sound, vibration, and pop-up screen): 1. Reminder to take a walk break; 2. Reminder of the right posture; 3. Reminder of the stretching exercises; 4. Reminder of the home-based exercises	Reminder	NR	4.88
Lo et al [38], inconsistent	1. Physical exercise program; 2. Educational material *pushed* to users through a social media platform	Recommended exercise duration: 20-30 minutes per day	Points-based rewards system to promote engagement with the app; reminder functions (daily)	Gamification and reminder	Time spent on exercises: mean 25 (SD 4) minutes per day; time spent on reading educational materials: mean 15 minutes per day (SD 14)	NR
Huber et al [35], 12 weeks	1. Physiotherapy and physical exercise; 2. Back pain–specific education; 3. Mindfulness and relaxation techniques	Daily content consists of components 1-3, up to 5 exercises per day	Chat function connects user with a coach to receive motivation and help	Motivation	NR	82.2
Clement et al [36], 24 weeks	RE^e^; additional component (4): Increased pool of each of the different exercise types (subdivided into 19 different difficulty levels in version 1.4 instead of 3 levels)	RE	RE	RE	Physical exercises: V1^i^: mean 1.99 (SD 1.61); V2^i^: mean 3.15 (SD 1.72); mindfulness exercises: V1: mean 1.36 (SD 1.43); V2: mean 2.42 (SD 1.82); educational content: V1: mean 1.51 (SD 1.42); V2: mean 2.71 (SD 1.89)	64.9
Priebe et al [37], 12 weeks	1. Physiotherapy and physical exercise; 2. Back pain–specific education; 3. Mindfulness and relaxation techniques	Daily content consists of components 1-3, up to 5 exercises per day	Feedback (smileys and congratulatory messages) for achieving improvements, health coach (chat function), and push-up reminders^e^	Motivation and reminders	Mean number of days the app was used: V1: mean 22.11 days^f,j^ (SD 10.56); V2: mean 30.92 days (SD 32.27)	V1: 82; V2: 62
Sandal et al [39], 6 weeks	1. General physical activity; 2. Strength and flexibility exercises; 3. Patient education (access to variety of tools and information for low back pain)	Daily goal: 10,000 steps; 4 weekly exercise sessions; 1 reading task on education	Weekly tailored self-management plans: 1. Suggest activity goals; 2. Suggest a new exercise program; 3. Suggest new education sessions	Multiple BCTs	After 6 weeks; mean values 65 app visits (range 1-188); 134 minutes spent on the app (range 0-889); visited the app on 22 of the 42 possible days (range 1-42)	13.72

^a^BCT: behavior change technique.

^b^At final follow-up measurement of the intervention group.

^c^Defined as completing at least one exercise session or reading 1 educational paper in weeks 9-12.

^d^RE: reported elsewhere; see Toelle et al [31] and Huber et al [35].

^e^Involves studies including the Kaia app; all information on the type of therapeutic components and applied interventions was extracted as described within the respective publication.

^f^Number of days within the whole intervention length.

^g^Those who completed ≥5 training sessions each week were considered high adherence, 3-5 training sessions medium adherence, and ≤2 training sessions low adherence.

^h^NR: Not reported.

^i^Comparison between 2 subsequent app versions: version 0.x (V1) and version 1.x (V2). V1 includes users who signed in before May 1, 2017, and V2 includes users who signed in after that date.

^j^A day was classiﬁed as an active day when the user logged into the app and completed at least one module.

### Personalized Decision Support Interventions

In all included studies, different kinds of decision support interventions were deployed to guide and accompany the user through the DTC program and to increase engagement with the app. Basic reminders in the form of push notifications were implemented most often [29-33,37,38], followed by a health coach chat function for motivational and reinforcing purposes [28,31,34-37], peer-group support through interactive discussion feeds or forums [28,30,34], a points-based rewards system [30,38], feedback messages after achieving improvements [37], and a tailored self-management plan that prompted suggestions on personalized activity goals and education sessions [39]. The applied decision support interventions encompassed a broad spectrum of behavior change techniques, including reminders, peer support, motivational messages, goal setting, coping methods, and gamification.

### User Retention and Attrition Rates

Overall, user retention with regard to the DTC app was mentioned in 75% (9/12) of the studies [25-39]. Of these 9 studies, 7 (78%) reported predominantly high engagement levels [28-31,34,36,38]. However, the reporting metrics were highly heterogeneous, with unclear relation to, and association with, the prerecommended time and app use frequency. Of these 9 studies, 2 (22%) [34,36] calculated the mean number of completed units per week, 2 (22%) [31,37] reported the mean number of days the app was used over the whole study duration, 1 (11%) [28] reported the mean number of days the respective therapeutic component was used, 1 (11%) [30] reported the mean number of completed therapeutic modules over the whole study duration, 1 (11%) [38] reported the mean number of minutes per day spent on the respective therapeutic modules, and 1 (11%) [39] reported the total number of app visits and the number of minutes spent in the app. The remaining study categorized engagement in high- to low-adherence groups based on the number of weekly training sessions [29]. The exact numbers with regard to user retention and engagement are presented in Table 4. Attrition rates ranging from 2.15% to 82.2% were reported in 92% (11/12) of the studies. In most studies [28-31,34], the attrition rates varied between 20% and 28%. Remarkably, in the studies with the lowest attrition rates [32,33,39], an RCT or prospective trial was conducted. In contrast, the studies with the highest attrition rates [35-37] were based on real-world evidence and retrospective app user–generated data analysis.

### Impact of DTC Apps

The impact of DTC apps and add-on decision support interventions was evaluated by considering the primary outcomes of pain and functional disability. In the included studies, the level of pain was measured using the Visual Analog Scale, the Numeric Rating Scale, and the Modified von Korff Pain Scale. The level of functional disability was measured using the Modified von Korff Disability Scale, the Roland Morris Disability Questionnaire, the Oswestry Disability Index, and the Modified Oswestry Disability Index. In 33% (4/12) [29,30,32,33] of the studies, both pain and functional disability were measured. In 67% (8/12) [28,31,34-38] of the studies, only pain levels were reported using the Numeric Rating Scale or Visual Analog Scale, and in 8% (1/12) [39] of the studies, only the functional outcome was measured using the Roland Morris Disability Questionnaire. Overall, in all included studies, there was a positive care effect in the DTC intervention group compared with baseline values, that is, in lower pain levels and increased functionality. A between-group comparison within 67% (8/12) of the studies revealed no significant difference in pain levels in 2 RCTs [31,32]. It should be noted that in some studies [21,24,34,39], participants had ongoing access to treatment as usual in addition to the DTC app, which was not described in detail. The results of the primary outcome measures and the respective treatment groups are presented in Table 5.

**Table 5:** Treatment groups and primary outcome results of the included studies (N=12).

Study	Intervention group	Control group	Primary outcome results^a^
Bailey et al [34]	Hinge Health Digital Care Program, including a new tablet, 2 Bluetooth wearable motion sensors, and one-on-one remote health coaching; treatment as usual	No control group	VAS^b^ ↑^c^
Priebe et al [28]	GP^d^-centered LBP^e^ treatment: 1. Electronic case report form; 2. Treatment algorithm for guideline-based clinical decision-making of GPs; 3. Teleconsultation between GPs and pain specialists for patients at risk for chronic back pain; 4. *Kaia* app	Treatment as usual with consideration of the *National guideline for the treatment of nonspeciﬁc back pain*	NRS^f^ ↑ (↑)^g^
Hou et al [29]	Patients with LBP who underwent lumbar spinal surgery were provided with a mobile phone–based eHealth program app as part of their rehabilitation program	Nonspecific usual care rehabilitation treatment	VAS ↑ (↑); ODI^h^ ↑ (↑)
Shebib et al [30]	Hinge Health Digital Care Program, including a new tablet, 2 Bluetooth wearable motion sensors, and one-on-one remote health coaching; treatment as usual	A total of 3 digital education articles from the digital care program; treatment as usual	MvK^i^ (pain) ↑ (↑); MvK (disability) ↑ (↑); ODI ↑ (↑)
Toelle et al [31]	Provided with the *Kaia* app	A total of 6 individual physiotherapy sessions over 6 weeks and high-quality web-based education, including motivating messages	NRS ↑ (↔^j^)^g^
Chhabra et al [32]	Provided with the *Snapcare* app; written prescription from the physician (see *Control group*)	Participants received a written prescription from the physician listing the prescribed medicines and dosage and stating the recommended level of physical activity	NRS ↑ (↔); MODI^k^ ↑ (↑)
Almhdawi et al [33]	Provided with the *Relieve my back* app; treatment as usual	Control group received a *placebo* version of the same app that included only advice about general nutrition and daily notifications with nutritional facts; treatment as usual	VAS ↑ (↑); ODI ↑ (↑)
Lo et al [38]	Retrospective evaluation study of the artificial intelligence–embedded *Well Health* app	No control group	NRS ↑
Huber et al [35]	Retrospective analysis of user data: *Kaia* app users who signed up before March 2017	No control group	NRS ↑
Clement et al [36]	Retrospective analysis of user data: *Kaia* app users who signed up on or after May 1, 2017	*Kaia* app users who signed up before May 1, 2017	NRS ↑ (↑)
Priebe et al [37]	Retrospective analysis of user data	*Kaia* app users who signed up before March 2017	NRS ↑ (↑)
Sandal et al [39]	Provided with the *selfBACK* app; treatment as usual	No control group	RMDQ^l^ ↑

^a^Main result of the intervention group after the last measurement in the study.

^b^VAS: Visual Analog Scale.

^c^Intervention had positive effect compared with baseline measurement.

^d^GP: general practitioner.

^e^LBP: low back pain.

^f^NRS: Numeric Rating Scale.

^g^Between-group differences are reported in parentheses.

^h^ODI: Oswestry Disability Index.

^i^MvK: Modified von Korff Scale.

^j^No difference in outcome.

^k^MODI: Modified Oswestry Disability Index.

^l^RMDQ: Roland Morris Disability Questionnaire.

Regarding adverse health events, in 75% (9/12) of the studies, no evidence of harm was reported after the implementation of DTC. Participants in a study [33] reported temporary discomfort; in another study [31], a patient was diagnosed with a lumbar disk herniation, which was declared an incidental finding. In the remaining study [29], 9 patients reported mostly mild, self-limited joint and back pain; of note, the patients underwent spinal surgery before starting the DTC. We have presented the results of additional secondary outcome measures, the time and frequency of measurements, and the mode of administration of surveys in Multimedia Appendix 2 [28-39].

## Discussion

### Principal Findings

This systematic review investigated the efficacy and effectiveness of DTC and add-on decision support interventions for people with LBP. Our analysis shows that all included studies observed positive health effects in the intervention group compared with baseline measures. In 67% (4/6) of the RCTs, between-group analysis indicated superior primary outcomes of the DTC program. Moreover, different DTC apps proved to have potentially significant benefits for particular cohorts. In a study [29], patients who had undergone spinal surgery shortly before starting the DTC and did not live close to the clinic received the DTC app as part of a remote rehabilitation program. Another study [33] explicitly targeted office workers aged 30-55 years to investigate the benefits of a digital care app on quality of life and functionality at work. In another study [28], the researchers aimed to prevent the development of chronic LBP by stratifying patients classified as high risk based on the STarT Back questionnaire through a general practitioner and, thus, providing them with a DTC app as early as possible to prevent a worsening condition [37,40]. Overall, no evidence of harm was reported, except for mild pain and a presumably incidental finding.

Notably, these results must be interpreted with caution when considering that 33% (4/12) of the studies did not include a control group, and in 63% (5/8) of the studies that included a control group, it did not have recommended treatment according to current clinical guidelines. Most trials included small to medium sample sizes, which applies to 67% (8/12) [29-33,35,38,39] of the studies with <200 participants and 42% (5/12) [29,31-33,39] of the studies with <85 participants in the intervention group. Overall, the study population was mostly homogeneous, with predominantly female, young to middle-aged participants of normal to moderate weight, limiting the transferability of the studies’ outcomes to other patient cohorts. The lack of long-term follow-up is another limitation in 83% (10/12) [28,30-35,37-39] of the studies. Moreover, overall user engagement and retention rates were reported to be medium to high, which we cannot ascertain in some cases because of unclear reporting. For instance, some studies reported their overall retention rates based on the mean days on which the participant completed at least one module or based on completing at least one therapy module in the last 3 weeks of the study, both of which are not attributable either to perpetual engagement or the use of differentiated key therapeutic components [34,37]. This adds to the difficulty of objectively measuring the actual number of completed therapeutic modules. To circumvent these challenges as well as the self-reporting biases, some DTC apps take advantage of wearable motion sensors or use an analytics platform to track interaction with the app [34,39].

Add-on decision support interventions accompanied DTC to enhance digital treatment by increasing user engagement and self-management capabilities in all investigated apps. Nonetheless, in most of the studies, rather basic rule-based decision support interventions were implemented, such as alert reminders or similar motivational push notifications. A more advanced data-driven recommendation system based on machine-learning was reported in a single study [38]. Research on data-driven support interventions has already demonstrated higher retention rates and increased user satisfaction in the self-management of LBP [41,42]. Therefore, implementing more complex decision support interventions is essential for achieving sustainable behavior change and high user engagement over a longer period, especially in a noncontrolled real-life environment.

In this review, it was not feasible to appraise the direct impact of either the single DTC key components, for example, exercise, educational material, or psychosocial content, or the decision support interventions, for example, peer support, on the primary health outcomes. Subsequently, it remains unclear to what extent DTC needs to be prescribed to achieve a marginal positive health effect for individual patient cohorts in terms of duration and number of exercise or education modules. In this regard, the effectiveness of DTC apps on the distinct subgroups of patients with LBP stratified according to acute, subacute, or chronic pain levels remains unclear and requires further subgroup-specific research. Despite overall positive findings, our assessment of the methodological quality revealed that the risk of bias in the included studies was moderate to high, especially in the nonrandomized trials.

### Correlation Among Retention, Attrition, and Health Outcome

A major unresolved research endeavor deals with the correlation between engagement levels in a DTC program and improvements in health-related outcomes. The studies in which this effect was examined more closely reported positive as well as negative findings. A positive correlation between higher user retention and a significantly better health outcome was found in 25% (3/12) [29,34,35] of the studies. In contrast, another 25% (3/12) [28,31,36] of the studies also concluded that there was no correlation between app use frequency and improved pain level or functional disability. The underlying rationale for participants to stay with the program or choose to discontinue is yet unknown and could be multidimensional. For instance, depending on whether a participant experiences sudden or early improvement in pain levels can be a driving factor for the decision to either quit or continue to reinforce the positive outcome [28]. Nonetheless, these contrary and contraintuitive findings should be analyzed in future trials by monitoring primary outcome levels more frequently and collecting valuable feedback from participants. This demand is also associated with the ongoing need for consistent reporting of user retention and attrition rates. The use of standardized metrics for subjective and objective use of DTC apps is necessary to gain more insights and enhance the comparability of studies [11].

Another interesting observation in this review is the divergence of attrition rates when comparing RCTs and retrospective evaluation studies, which specifically consider people who have downloaded the DTC app on their own initiative. The lowest attrition rates were observed in 2 RCTs [32,33] and a prospective trial [39]. In contrast, the studies [35-37] with the highest attrition rates were based on real-world evidence and retrospective user-generated data analysis. One apparent reason for low attrition might have been the user’s awareness of being part of a trial or the participant’s compensation for the RCT, which involved vouchers, money, or free access to the app after the conclusion of the study. In contrast, participants who self-reportedly downloaded the app and eventually also paid for it on a monthly basis tended to quit the program earlier. Despite the fact that this observation was not adjusted based on the varying number of participants or the duration of the intervention, it shows that retrospective studies based on real-world evidence possibly provide insights into the real-life adoption and use of DTC apps [12]. In fact, in future data-driven research on digital health interventions, the analysis of homogeneous and structured data related to engagement and self-reported outcome measures could further advocate retrospective cohort evaluation studies. Data obtained from users who have downloaded a DTC app either on their own initiative or after receiving a physician’s prescription could be provided to research platforms and databases and, thus, facilitate the evaluation of real-life adoption and effectiveness. These benefits and the quickly evolving regulatory environment in favor of digital ecosystems in the EU, such as the EU-funded Smart4Health project, underline the relevance and timeliness of this review’s approach [43].

### Rising Uptrend of DTC App-Based Clinical Trials

We found additional studies investigating the benefits of divergent internet interventions or apps to support digital treatment of LBP during our search process. For instance, we identified a study involving an app that enables continuous pain monitoring for people with LBP [44], a study investigating the use of a website to support people in their self-management of LBP [45], a study that aimed to examine the benefits of a DTC app on the depressive disorder in patients with LBP [46], and a publication that describes 2 case studies in which a virtual reality system delivers functional rehabilitation exercises to people with LBP [47]. Moreover, we found several other research projects investigating their app-based therapeutic programs at an early stage of their development in the form of proof-of-concept, qualitative acceptability studies or research protocols [18,48-52]. This underlines our observation with regard to the exponential rise of clinical trials concerning DTC and decision support apps in the past 5 years.

### Limitations

This systematic review includes some limitations. First, we only considered English- and German-language literature, which might have led to excluding other potential eligible studies. Moreover, we only included LBP-related studies and excluded those investigating DTC apps for other similar health conditions, for instance, neck pain, shoulder pain, or musculoskeletal pain in general. Another limitation is the validity of this review with regard to the level of evidence. We are aware that systematic reviews that include only RCTs provide the highest level of evidence; however, considering studies based on real-world user data as well turned out to be a feasible approach, which we consider inevitable for future systematic reviews of digital health app trials.

Furthermore, although most of the included studies in this review reported overall positive health effects, we are cautious about deriving any clinical implications based on our findings. Because of the explorative approach that involved waiving study preregistration, not including traditional search terms such as *eHealth* and *mHealth*, and the fact that we focused on studies published from March 1, 2016, to October 15, 2020, we cannot exclude a variety of biases that may have occurred. Therefore, we have refrained from providing essential clinical recommendations for regulatory decisions and do not recommend copying this search strategy, which supported the specific objective of this review exclusively. The aim of this paper is to evaluate recently published clinical evidence regarding the efficacy and effectiveness of digital therapeutic interventions for people with LBP. However, DTC apps, including the broad range of implemented decision support interventions, experience continual improvements with new features and amendments concerning both front-end and back-end of an app. These advancements require ongoing clinical trial–based evaluations regarding their impact on health outcomes, user retention, and attrition rates, especially in this new field of digital therapy. Further research is needed to clarify whether DTC apps are so unique that they need to be evaluated individually or clinical implications can be made based on an overarching systematic review.

### Conclusions

This systematic review demonstrates the benefits of DTC for people with LBP with regard to both primary outcomes of pain and functional disability. There is also evidence that decision support interventions benefit overall engagement with the app and increase participants’ ability to self-manage their recovery process. However, because of mostly homogeneous study populations and the unclear correlation between user retention and improvements in primary outcomes, no general conclusion can be drawn either on the optimal intervention duration or the required number of exercise modules for individual cohorts. Finally, including retrospective evaluation studies of real-word user-generated data in future systematic reviews of digital health app trials can reveal new insights into the benefits, challenges, and real-life adoption of DTC programs.

## Appendix

Multimedia Appendix 1 Search queries.

Multimedia Appendix 2 Results of all reported primary and secondary outcome measures.

## Abbreviations

DTC: digital therapeutic care
EU: European Union
LBP: low back pain
PRISMA: Preferred Reporting Items for Systematic Reviews and Meta-Analyses
RCT: randomized controlled trial
ROB 2: Risk of Bias 2
ROBINS-I: Risk of Bias in Non-Randomized Studies of Interventions
