# Supplementary material for: Digital Therapeutic Care and Decision Support Interventions for People With Low Back Pain: Systematic Review
Source: JMIR Rehabil Assist Technol. 2021 Nov 19;8(4):e26612. doi: 10.2196/26612 (PMC8663573; doi:10.2196/26612)
Supplement: Multimedia Appendix 1 [file rehab_v8i4e26612_app1.docx]

**Search Queries**

Medline (via PubMed)

("back pain" OR "low back pain"[MeSH] OR "lower back pain" OR "LBP" OR Low Back Pain / therapy*[MeSH] OR "chronic LBP" OR "acute LBP" OR "lumbago" OR "lumbar spine" OR "lumbar pain" OR "sciatica" ) AND ("digital therapeutic" OR "digital care" OR "personalized decision support" OR "personalized health service" OR "digital support" OR "digital intervention" OR "digital care" OR "app" OR "mobile applications"[MeSH] OR "online intervention" OR "internet intervention" OR "web-based" OR "digital treatment" OR "online treatment" OR "smartphone" OR "Internet-Based Intervention" OR "Self-Management" OR "telemedicine" OR “decision support”)

Cochrane Library, Web of Science

("back pain" OR "low back pain" OR "lower back pain" OR "LBP" OR "chronic LBP" OR "acute LBP" OR "lumbago" OR "lumbar spine" OR "lumbar pain" OR "sciatica") AND ("digital therapeutic" OR "digital care" OR "personalized decision support" OR "personalized health service" OR "digital support" OR "digital intervention" OR "digital care" OR "app" OR "mobile applications" OR "online intervention" OR "internet intervention" OR "web-based" OR "digital treatment" OR "online treatment" OR "smartphone" OR "Internet-Based Intervention" OR "Self-Management" OR "telemedicine" OR “decision support”)

PEDro (Physiotherapy Evidence Database)

Abstract & title: Digital therapeutic, digital care, digital treatment, smartphone, app, decision support, internet intervention, online intervention. Problem: Pain.
