# Supplementary material for: Digital Therapeutic Care and Decision Support Interventions for People With Low Back Pain: Systematic Review
Source: JMIR Rehabil Assist Technol. 2021 Nov 19;8(4):e26612. doi: 10.2196/26612 (PMC8663573; doi:10.2196/26612)
Supplement: Multimedia Appendix 2 [file rehab_v8i4e26612_app2.docx]

**Results of all reported primary and secondary outcome measures.**

| Reference | All outcome measures^c^ | Measurement –  time and frequency | Mode of measurement | Primary and secondary  outcome results ^a,b,c^ |
| --- | --- | --- | --- | --- |
| Bailey et al [34] | 1. VAS  2. PHQ-9  3.GAD-7  4. One-year surgery likelihood  5. WPAI  6. MvK | 1. Weekly after an ET session; or unprompted, for a total of up to 2 pain scores per week.  2.-6. At baseline, 6-weeks, and 12-weeks. | In-app survey | 1. VAS ↑  2. PHQ-9 ↑  3. GAD-7 ↑  4. Surgery likelihood ↑  5. WPAI ↑  6. MvK ↑ |
| Priebe et al (a) [28] | 1. NRS  2. DASS  3. HFAQ  4. VR-12  5. GCPS | At baseline and 3 months follow up | At baseline:  I: Tablet  C: Paper/pencil  At follow-up:  via email | 1. NRS ↑ (↑)  2. DASS ↑  3. HFAQ ↑  4. VR-12 ↑  5. GCPS ↑ |
| Hou et al [29] | 1. VAS  2. ODI  3. EuroQol-5D  4. SF-36  5. QoL Likert-scale | Preoperatively, and at  3, 6, 12, and 24 months postoperatively | Paper based surveys | 1. VAS ↑ (↑)  2. ODI ↑ (↑)  3. EuroQol-5D ↑  4. SF-36 ↑  5. Likert scales ↑ |
| Shebib et al [30] | 1. MvK (pain)  2. MvK (disability)  3. ODI  4. VAS  5. VAS  6. Surgery intent  7. How well do you understand your condition? | 1.,2.: At screening, week 4, week 8, and week 11  3.: At baseline, week 11  4.-7.: baseline, week 12  Each two times per week | In-app survey | 1. MvK (pain) ↑ (↑)  2. MvK (disability) ↑ (↑)  3. ODI ↑ (↑)  4. VAS (pain) ↑ (↑)  5. VAS (daily life impact) ↑ (↑)  6. Surgery intent ↑ (↑)  7. Understand condition ↑ (↑) |
| Toelle et al [31] | 1. NRS  2. HFAQ (functionality)  3. GCPC  3. VR-12  4. R-12 (physical component) | 1. At the end of each day of therapy;  1.-4.: At baseline, 6 weeks, and 12 weeks | 1. Via an in-app pain diary  2.-4.: Via postal service | 1. NRS ↑ (↔)  2. HFAQ (functionality) ↔ (↔)  3. GCPC ↔ (↔)  3. VR-12 (mental) ↑ (↔)  4. VR-12 (physical) ↑ (↔) |
| Chhabra et al [32] | 1. NRS  2. MODI  3. Daily physical activity  4. CSS - Current Symptom Score | At baseline and 12 weeks | In person and via telephone interview | 1. NRS ↑ (↔)  2. MODI ↑ (↑)  3. Physical activity ↑  4. CSS ↑ |
| Almhdawi et al [33] | 1. VAS  2. ODI  3. SF-12  4. DASS  5. Pittsburgh Sleep Quality Index  6.IPAC  7. ‘Google Play’ firebase logs | At baseline and 6 weeks | Unclear  (“by evaluators”) | 1. VAS ↑ (↑)  2. ODI ↑ (↑)  3. SF-12 ↑  4. DASS ↔  5. Pittsburgh Sleep QI ↔  6. IPAC ↔  7. ‘Google Play’ firebase logs ↑ |
| Lo et al [38] | 1. NRS  2. Time spent on ET  3. Usage of other conservative interventions while using Well Health app | At baseline | Self-reported evaluation questionnaire | 1. NRS ↑  2. Time spent on ET ↑  3. Usage of other interventions ↑ |
| Huber et al [35] | 1. NRS | At the end of each day of therapy in a pain diary | In-app user reported pain levels | 1. NRS ↑ |
| Clement et al [36] | 1. NRS  2. Duration of activity  3. Number of completed exercises | At the beginning of each day of therapy in a pain diary | In-app user reported / usage data | 1. NRS ↑ (↑)  2. Activity duration (↔)  3. Completed exercises (↑) |
| Priebe et al (b) [37] | 1. NRS | Daily – after log-in | In-app user reported pain levels | 1. NRS ↑ (↑) |
| Sandal et al [39] | 1. RMDQ  2. FABQ  3. PSFS  4. PSEQ  5. QoL  6. VAS  7. BIPQ | Baseline vs. 6 weeks | Web-based questionnaire | 1. RMDQ ↑ |

^a^ Main results of the intervention group after last measurement

^b^ Visualization of results: ↑ Intervention has positive effect compared to baseline measurement; (compared to control group);

↓ Intervention has negative effect; ↔ no difference in outcome; ; (between-group differences are reported in brackets).

^c^ Abbreviations: VAS: Visual Analog Score; EQ-5D: EuroQol 5-Dimension Health Questionnaire; ODI: Oswestry Disability Index; MODI: Modified Oswestry Disability Index; SF-36: 36-item Short-Form Health Survey; SF-36 GH: General health for 36-item Short-Form Health Survey; SF-36 PF: Physical functioning for 36-item Short-Form Health Survey; PHQ-9: patient health questionnaire 9-item scale; GAD-7: generalized anxiety disorder 7-item scale; WPAI: work productivity and activity impairment; MvK: Modified von Korff – scale; NRS: 11-point numeric ratings scale; DASS: Depression-Anxiety-Stress-Scale; HFAQ: Hannover Functional Ability Questionnaire; VR-12: Veterans RAND12 Item Health Survey; GCPS: Graded Chronic Pain Status; IPAQ: International Physical Activity Questionnaire; FABQ: Fear Avoidance Belief Questionnaire; PSFS: Patient Specific Function Scale; PSEQ: Pain Self-Efficacy Questionnaire: PHQ: Patient Health Questionnaire; PSS: Perceived Stress Scale; BIPQ: Brief Illness Perception Questionnaire.
